# Insights into the Structural-Transport Property Relationship of the Size-Dependent Organic Molecules on Metal Surfaces

**DOI:** 10.3390/molecules31213848

**Published:** 2026-10-25

**Authors:** Guirong Su, Yong Fan, Shuguang Zhang, Yonghao Zhao

**Affiliations:** 1 Jiangsu Provincial Engineering Research Center for Structure-Function Integrated Metallic Materials for Harsh Environments, School of Materials Science and Engineering, Hohai University, Changzhou 213200, China; shuguang0502@163.com; 2 Innovation Center for Critical Materials in Hydraulic Infrastructure Safety and Water Environment Restoration, School of Materials Science and Engineering, Hohai University, Changzhou 213200, China; 3 School of Intelligent Agricultural Equipment Engineering, Shandong University of Technology, Zibo 255000, China; fanyong@sdut.edu.cn

**Keywords:** density functional theory, metal/organic interface, energy-level alignment, Schottky barrier height, structural-transport property relationship

## Abstract

Organic semiconductors are pivotal for emerging flexible displays, large-area electronics, and low-cost photovoltaics, where device performance crucially hinges on energy-level alignment at metal/organic interfaces. However, the vast molecular and substrate design space hinders the development of predictive interface design rules. Here, we systematically investigate the adsorption and charge transport of size-dependent aromatic molecules across six transition metal surfaces with density functional theory (DFT) calculations. The interfacial interactions govern two distinct regimes: physisorption and chemisorption. Expanding molecular size drives a transition toward strong chemisorption, enhancing electronic coupling and charge transfer, which intensifies Fermi-level pinning and lowers the slope of Schottky barrier heights versus metal work functions. Moreover, using non-equilibrium Green’s function (NEGF) simulations, we formulate the interfacial descriptor, T, that integrates both the Schottky barrier height and tunneling barrier width. Remarkably, this descriptor correlates exponentially with device output current, achieving >90% predictive accuracy and significantly outperforming conventional Schottky models. This relationship remains robust upon heteroatom incorporation, demonstrating broad universality across diverse molecular electronic architectures.

## 1. Introduction

Propelled by Moore’s Law, the continuous scaling of silicon semiconductors has exponentially increased integration density, enhancing performance and cost-effectiveness [1,2,3]. However, approaching the sub-10 nm regime, this trajectory faces fundamental physical limits and pronounced quantum effects that fundamentally alter device physics [4,5]. Organic semiconductors—offering intrinsic flexibility, structural tunability, low cost, and large-area processability—have thus emerged as promising alternatives [6,7], for organic light-emitting diodes (OLEDs) [8], organic photovoltaics [9], organic field-effect transistors [10], and molecular switches [11]. In these devices, metal electrodes serve as critical contacts for charge injection and extraction, dictating overall electrical, optical, and transport performance [12,13]. To fulfill specific functionalities, stringent and distinct selection criteria for metals and organic molecules are required across different devices, as well as between different components within the same device [14,15]. For instance, in OLEDs, high-work-function materials are strategically selected for the anode to facilitate hole injection, whereas low-work-function materials are favored at the cathode to bolster electron injection efficiency [16,17]. Consequently, tailoring interfacial structures and optimizing transport properties across metal/organic junctions are paramount for advancing high-performance organic electronics.

This demand highlights the necessity of meticulously designing organic molecular architectures and matching them with appropriate metal contacts. However, given the vast diversity of both organic molecules and metals [18,19,20], their potential combinations are virtually limitless, often trapping the interfacial design paradigm in inefficient trial-and-error iterations. To overcome this bottleneck, establishing the fundamental relationship between interfacial structure and transport performance is imperative [21,22]. Currently, this endeavor faces two major challenges: (i) The metal/organic interface is governed by multifaceted and intricate interactions, including covalent bonding, van der Waals forces, hydrogen bonding, charge transfer, and Pauli repulsion [23,24]. Consequently, simply comparing pristine metal properties (e.g., work function) with intrinsic molecular levels—such as electron affinity (EA) and ionization potential (IP)—frequently fails to accurately predict charge injection and extraction barriers [25,26]. (ii) Experimentally measured currents inherently reflect the collective behavior of the complete metal/molecule/metal junction rather than an isolated metal/molecule interface. This systemic response obscures the direct correlation between key interfacial parameters (e.g., work function and Schottky barrier) and transport current. As a result, a universal and reliable descriptor capable of bridging metal/organic interfacial characteristics with macroscopic transport behavior remains elusive.

**Figure 1:**
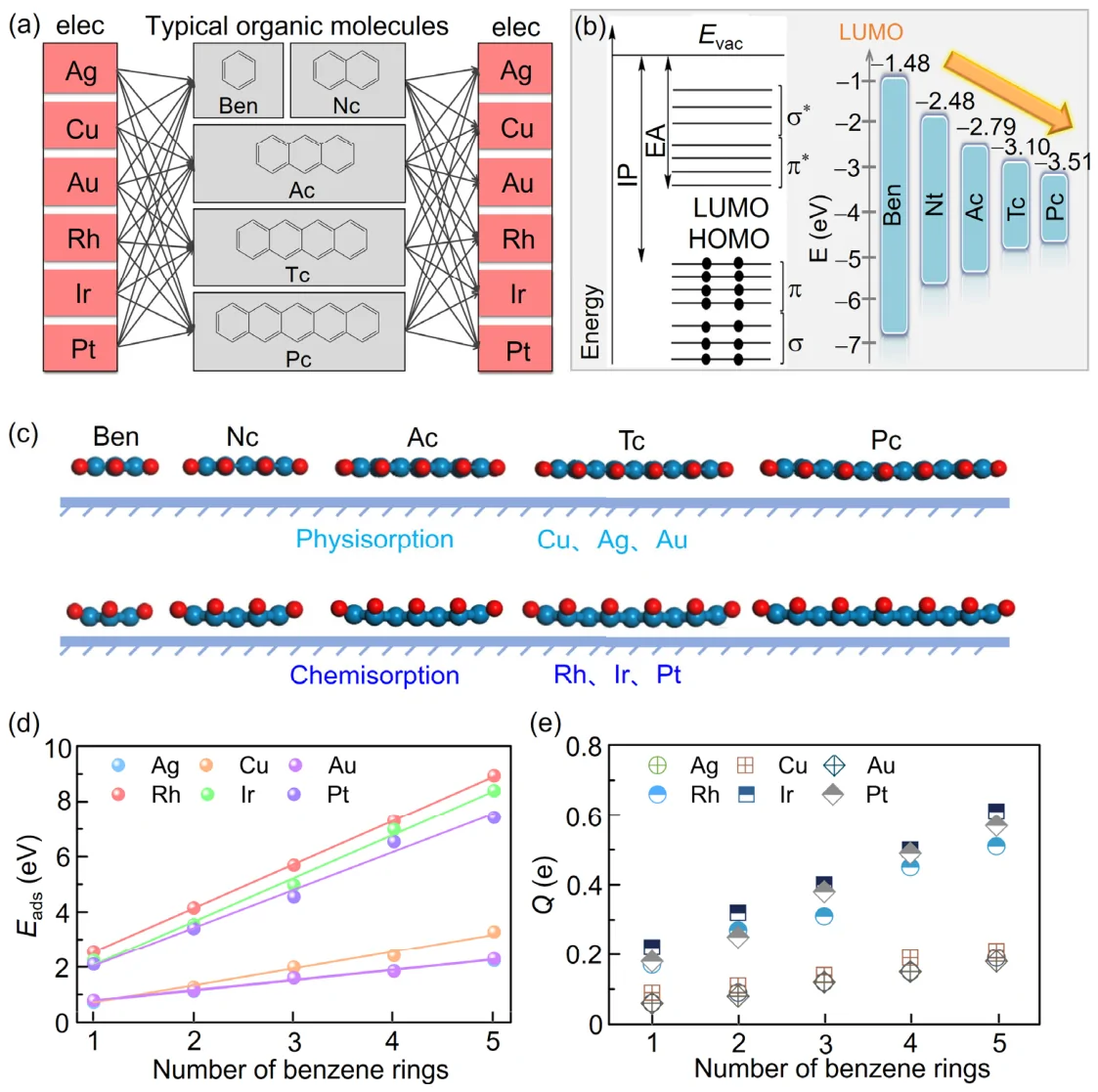
(**a**) Schematic illustration of metal/molecule/metal junctions formed by various electrode materials and typical organic molecules: Ben, Nc, Ac, Tc, and Pc. (**b**) Electronic energy levels of a conjugated molecule. The right panel displays the calculated gas-phase energy levels of these molecules, showing a clear downward trend in the LUMO position with increasing molecular length. (**c**) Side views of organic molecules adsorbed on the (111) surfaces of various transition metals. The configurations fall into two distinct regimes: weak physisorption on noble metals (Cu, Ag, and Au) with negligible molecular deformation and strong chemisorption on reactive metals (Rh, Ir, and Pt) accompanied by pronounced bending. (**d**) Adsorption energy (*E*_ads_) and (**e**) amount of interfacial charge transfer (*Q*) as a function of the number of benzene rings on different metal substrates.

In this paper, we conduct a comprehensive theoretical investigation into the adsorption and electron transport characteristics of size-dependent acene molecules on six representative transition metal surfaces using van der Waals-inclusive density functional theory (DFT). Aromatic hydrocarbons—spanning benzene (Ben), naphthalene (Nc), anthracene (Ac), tetracene (Tc), and pentacene (Pc) as depicted in Figure 1a—serve as the foundational building blocks for the vast majority of functionalized organic semiconductors [27,28,29], rendering them ideal prototypical models for exploring organic/inorganic hybrid interfaces [30]. Based on the nature of interfacial interactions, the investigated metal/organic interfaces are systematically classified into physisorbed and chemisorbed regimes. We reveal that extending the molecular size significantly intensifies interfacial electronic coupling via enhanced orbital hybridization and charge transfer. This behavior promotes a more pronounced Fermi-level pinning effect, as evidenced by the progressively decreasing slope of the Schottky barrier height with respect to the metal work function. Combined with the non-equilibrium Green’s function (NEGF) transport calculation database, we further propose an interfacial descriptor T by concurrently accounting for thermal excitation and quantum tunneling. This descriptor exhibits an exponential relationship with the output current for these size-dependent molecular junctions in practical circuit environments, achieving a predictive accuracy exceeding 90% and markedly outperforming conventional Schottky theory. Notably, the trends remain valid even when more reactive heteroatoms are incorporated into the conjugated backbone, further demonstrating the universality of our descriptor in molecular junction design.

## 2. Results and Discussion

### 2.1. Molecular Models and Size-Dependent Electronic States

The vast majority of functional organic semiconductors are derived from the chemical modification or doping of fundamental polycyclic aromatic hydrocarbons (PAHs), such as Ben, Nc, Ac, Tc, and Pc. As quintessential linearly fused π-conjugated frameworks, these oligoacenes serve as benchmark prototypes for tailoring electronic states and understanding organic/inorganic hybrid interfaces [12,31,32,33]. Given their fundamental significance, we selected this homologous series to systematically elucidate interfacial coupling and structure–transport property relationships. Specifically, we constructed a comprehensive set of metal/molecule/metal junction models by pairing these five representative acenes with six transition metal electrodes (Ag, Cu, Au, Rh, Ir, and Pt) spanning diverse electronic coupling regimes, as schematically illustrated in Figure 1a.

As typical conjugated systems with alternating single and double bonds, their electronic structures are governed by the frontier molecular orbitals (Figure 1b, left panel). The highest occupied molecular orbital (HOMO) and lowest unoccupied molecular orbital (LUMO) exhibit dominant π and π^*^ characteristics, defining the molecular ionization potential (IP) and electron affinity (EA) with respect to the vacuum level (*E*_vac_) [12]. The absolute HOMO and LUMO energy levels were obtained from DFT-calculated density of states (DOS) profiles, as detailed in the Methods section. With the extension of the conjugated backbone from 1 to 5 benzene rings, the calculated gas-phase energy levels display a significant downward shift in the LUMO position—decreasing from −1.48 eV in benzene to −3.51 eV in pentacene (Figure 1b, right panel)—along with a monotonic narrowing of the HOMO–LUMO fundamental gap. This systematic modulation of frontier orbital alignment provides an ideal platform to elucidate how molecular length and electrode hybridization jointly govern the interfacial adsorption states and transport behaviors.

### 2.2. Interfacial Adsorption Behaviors and Charge Redistribution

To determine the stable adsorption geometries and interfacial interactions, we employed the PBE+vdW^surf^-R method to model organic molecules across various transition metal (111) surfaces, which has proven accurate across both physisorption and chemisorption regimes [34,35]. The (111) surface was chosen due to its minimal surface energy among fcc facets and its widespread prevalence as the benchmark substrate in experimental molecular electronics [36,37,38]. As illustrated in Figure 1c, the optimized configurations clearly classified into two distinct regimes: weak physisorption on noble metals (Cu, Ag, and Au) and strong chemisorption on reactive transition metals (Rh, Ir, and Pt). In the physisorption regime, the molecules predominantly adsorb at the hcp sites, with their C–C bonds forming a 30° angle with adjacent metal bonds (Figure S1). In this scenario, the molecules undergo minimal deformation and primarily assume a flat-lying state, which aligns with Scanning Tunneling Microscopy (STM) experimental observations of molecules freely diffusing across Cu(111) and Au(111) step edges [39,40]. Conversely, on the more reactive Pt(111) surface, Low-Energy Electron Diffraction (LEED) analysis revealed that the organic molecules undergo chemisorption at the bridge sites [41], leading to substantial molecular deformation.

The influence of variations in molecular size on binding strength is depicted in Figure 1d. For all substrates, the adsorption energy (*E*_ads_) scales linearly with the number of benzene rings (from Ben to Pc). Notably, the chemisorbed systems exhibit significantly higher binding energies compared to the physisorbed systems, and this energy disparity widens dramatically as the conjugated backbone elongates. Notably, in physisorbed systems, changes in molecular size lead to significantly higher adsorption energy on the Cu(111) surface compared to the Ag(111) and Au(111) surfaces. This finding hints at a potential shift for molecules on the Cu(111) surface, transitioning from physisorption to chemisorption [42].

To gain a deeper understanding of the changes in interfacial electronic property during the adsorption process, we further calculated the total transferred charges by subtracting the contributions of the isolated organic molecule and the pristine metal surface from the electron density distribution of the entire adsorption system (see Section 4 for more details). Upon adsorption, there is a depletion of electron density in the vicinity of the molecule side between the adsorbate and the substrate, while an accumulation occurs in the region adjacent to the substrate side (Figure S2). This observation clearly indicates a significant transfer of electrons (*Q*) from the molecule to the substrate (Figure S3). Crucially, the reactive chemisorption surfaces (Rh, Ir, Pt) display much larger charge transfer amounts (up to 0.61 e) compared to the weakly interacting noble metals (up to 0.21 e) (Figure 1e), demonstrating that the degree of chemical bonding dictates the magnitude of interfacial charge redistribution and dipole formation.

### 2.3. Interfacial Energy-Level Alignment and Fermi-Level Pinning Behaviors

**Figure 2:**
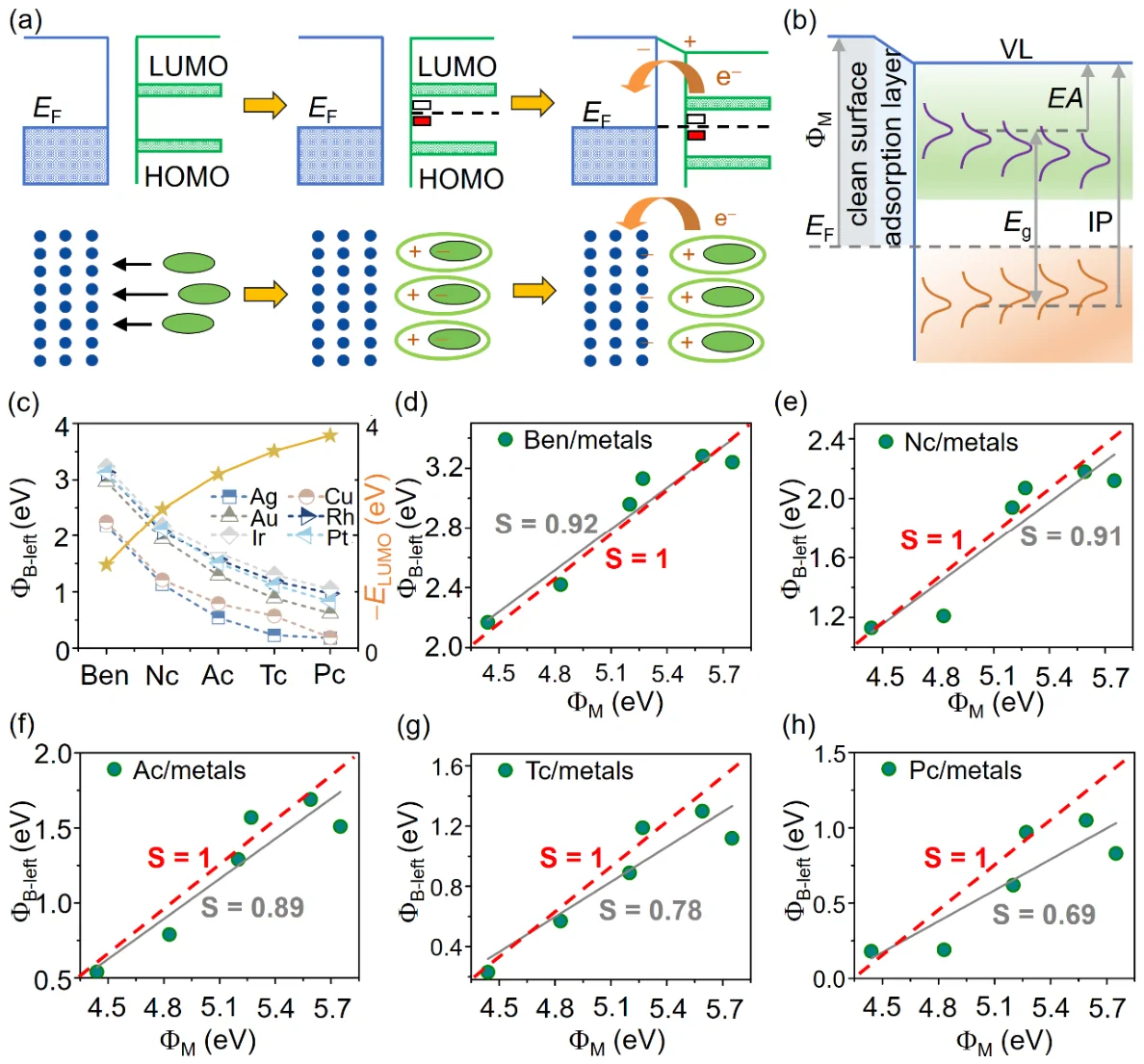
(**a**) Schematic illustration of the formation mechanism of the interfacial dipole at the metal/organic interface: isolated materials before contact (left), Fermi level pinning induced by emerging interface/gap states upon contact (middle), and charge transfer from the organic molecule to the metal establishing thermodynamic equilibrium (right). (**b**) Energy–level alignment at the metal/organic interface. (**c**) Schottky barrier heights of the left metal/organic interface (Φ_B-left_, left axis) and negative LUMO levels (−*E*_LUMO_, right axis) for molecules on metal surfaces. Each yellow star represents the LUMO energy at the respective abscissa positions of Ben, Nc, Ac, Tc and Pc. (**d**–**h**) Φ_B_ as a function of Φ_M_ for Ben, Nc, Ac, Tc, and Pc systems, respectively. The solid lines represent linear fits with pinning factors (S), while the red dashed lines denote the ideal Schottky–Mott limit (S = 1).

Extensive experimental and theoretical studies have established that the Schottky barrier height formed at the metal/organic interface (Φ_B-left_) serves as a decisive parameter dictating interfacial charge injection and device contact resistance [25,43]. As illustrated schematically in Figure 2a, when isolated materials are brought into contact, emerging gap/interface states induce Fermi-level pinning, driving spontaneous electron transfer from the organic adsorbate to the metal substrate until thermodynamic equilibrium is established. This interfacial charge redistribution, combined with the Pauli repulsion (push-back effect), creates an interface dipole that shifts the local vacuum level (VL) across the adsorption layer (Figure 2b). Consequently, Φ_B_ is collectively determined by:

Φ_B_ = Φ_M_ − *E*_LUMO_ − Φ_BD_ (1)

where Φ_M_ denotes the metal work function, *E*_LUMO_ is the molecular gas-phase LUMO energy level, and Φ_BD_ is the resulting magnitude of the interface dipole.

Based on these insights, we comprehensively examined the pristine work function and their shifts (ΔΦ_M_) with molecular adsorption and the decoupled contributions from molecular and interfacial dipoles. The computed clean work functions show excellent consistency with available experimental and theoretical literature [44]. By isolating the molecular dipole from the work function shifts, we determined the net interfacial dipole [45] (Table S1), revealing that chemisorption induces substantially larger molecular polarization than physisorption. Concurrently, for any selected metal substrate, the calculated Φ_B_ decreases monotonically as the π-conjugated backbone extends from benzene to pentacene (Figure 2c, left axis). This barrier reduction mirrors the progressive downward shift in the frontier LUMO level (−*E*_LUMO_, Figure 2c, right axis), establishing that orbital alignment via conjugated chain extension reliably modulates interfacial electron injection.

We further examined the substrate-dependent barrier modulation to quantify the degree of Fermi-level pinning across the oligoacene series (Figure 2d–h). By linearly correlating the Schottky barrier height (Φ_B-left_) with the pristine metal work function, the pinning parameter S = Φ_B_/Φ_M_ was extracted (S = 1 represents the unpinned Schottky–Mott limit). At the benzene interface (Figure 2d), the barrier exhibits a nearly ideal linear response with S = 0.92, indicating that Fermi-level pinning is negligible and vacuum-level alignment predominates. In contrast, extending the π-conjugation from naphthalene to pentacene causes S to decrease monotonically from 0.91 to 0.69 (Figure 2e–h). This pronounced departure from the ideal limit is driven by stronger metal/molecule hybridization, elevated interface state densities, and intensified interfacial charge transfer. These results provide crucial theoretical insights for optimizing carrier injection in organic nanoelectronics.

### 2.4. Structural-Transport Property Relationship

**Figure 3:**
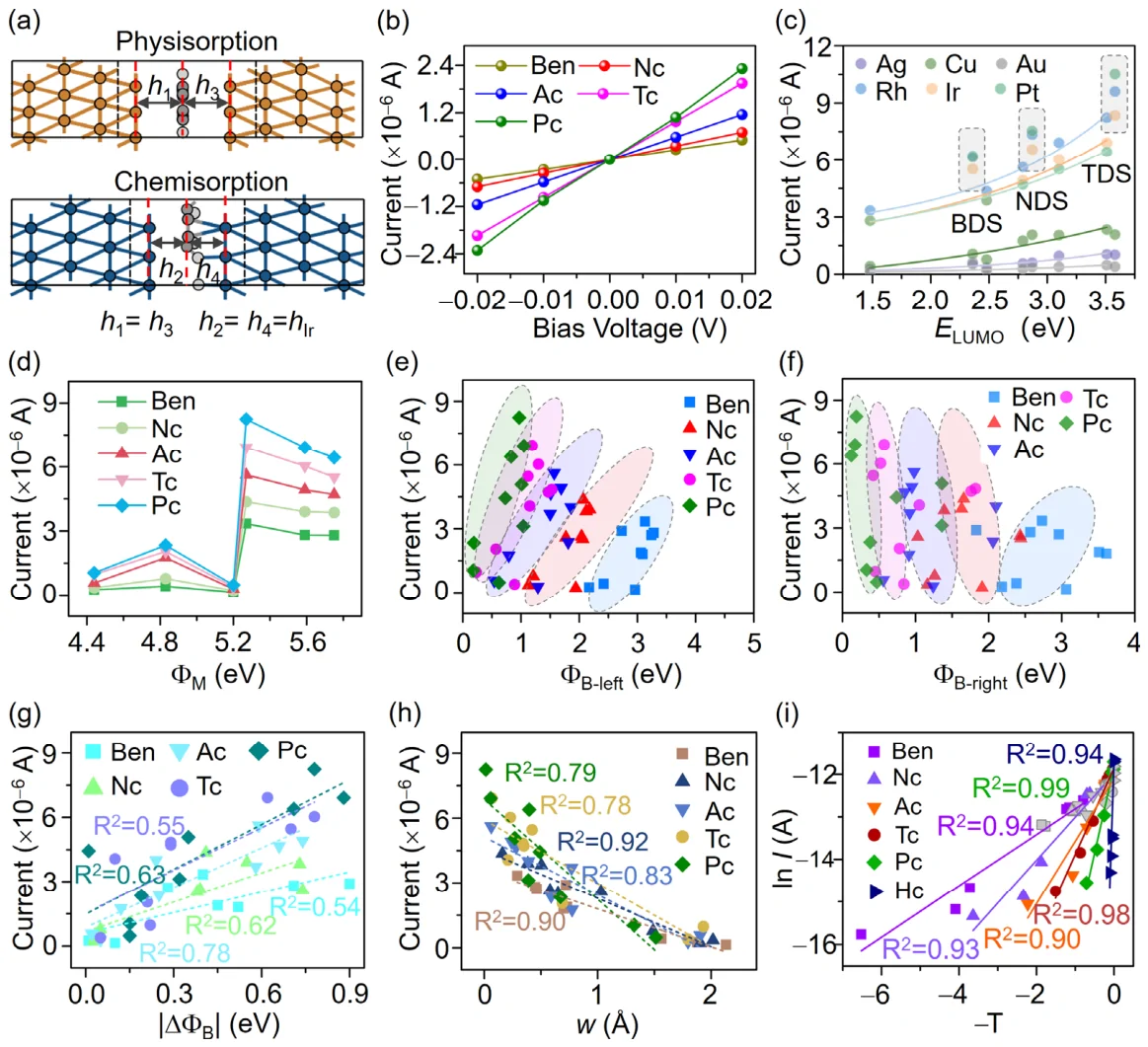
(**a**) Schematic configurations of molecular Schottky junctions under physisorption and chemisorption contact regimes in circuit models. (**b**) Current–voltage (I–V) characteristics of single-molecule junctions with various organics on Ag(111) electrodes. (**c**) Dependence of the junction current on the molecular LUMO energy level (*E*_LUMO_) under a bias voltage of 0.01 V across different electrode materials. (**d**) Output current as a function of metal work function. (**e**,**f**) Output current as a function of the Schottky barrier height at the left (Φ_B-left_) and right (Φ_B-right_) interfaces, respectively. (**g**) Linear scaling between the output current and the interfacial Schottky barrier difference (|ΔΦ_B_|). (**h**) Dependence of the junction current on the tunneling width at the right interface (*w*_right_). (**i**) Linear fitting between the natural logarithm of current (ln*I*) and the transmission parameter (−T), with corresponding R^2^ values indicated.

To systematically examine the electronic transport properties of the metal/organic interfaces in realistic circuit configurations, we further conducted non-equilibrium Green’s function combined with density functional theory (NEGF-DFT) calculations. As schematically illustrated in Figure 3a, two-probe metal/molecule/metal junction configurations were constructed under both physisorption and chemisorption contact regimes using independently constructed, fully relaxed interfacial geometries to guarantee realistic contact environments. In sharp contrast to the constant-height (uniform electrode separation) model adopted in the previous study [46], the electrode separations are symmetrically defined by *h*_1_ = *h*_3_, for physisorbed systems, corresponding to twice the equilibrium adsorption height of each respective interface. For chemisorbed systems with pronounced molecular buckling, the inter-electrode spacing is unified to *h*_2_ = *h*_4_ = *h*_Ir_ based on the maximum adsorption height found on Ir(111), providing a standardized platform to compare transport characteristics across different chemical interfaces.

The calculated current–voltage (I–V) characteristics for the Ag(111) junction series are presented in Figure 3b. Across the applied bias window, the output current increases monotonically with bias voltage while exhibiting negligible rectification behavior, which is primarily attributed to the nearly symmetric potential barriers at the left and right physisorption interfaces. Furthermore, the transmission current systematically scales up as the molecular conjugated backbone extends from Ben to Pc. This size-dependent enhancement aligns well with our Schottky barrier calculations in Figure 2c, corroborating that extended π-conjugation and the associated barrier reduction promote electronic conduction across the metal/molecule/metal junctions.

**Figure 4:**
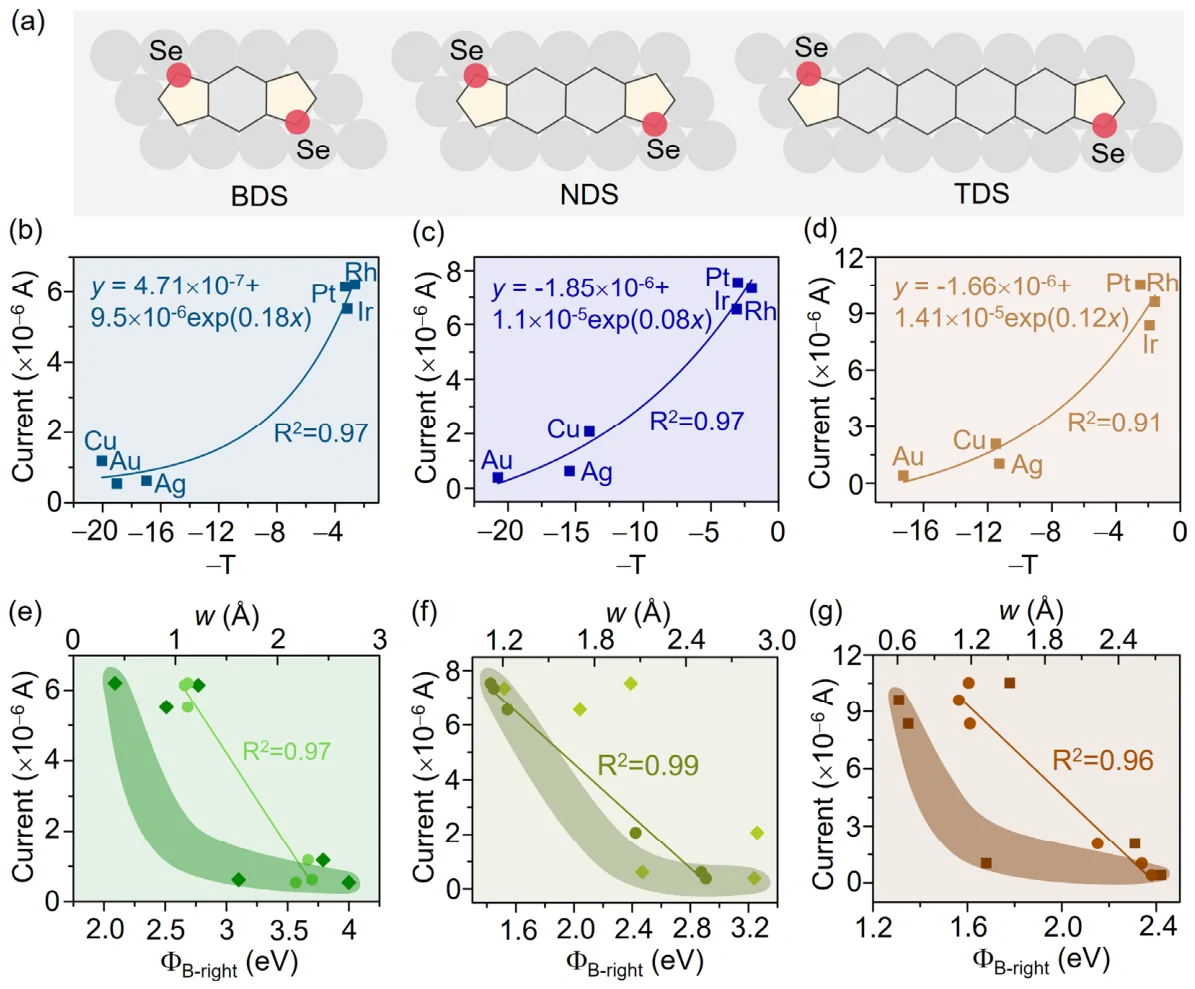
(**a**) Chemical structures of BDS, NDS, and TDS. (**b**–**d**) Dependence of the junction current on the transmission parameter (−T) across different metallic electrodes (Cu, Ag, Au, Pt, Ir, and Rh) for (**b**) BDS, (**c**) NDS, and (**d**) TDS, along with the corresponding exponential fitting curves. (**e**–**g**) Junction current plotted against the right-interface Schottky barrier height (Φ_B-right_, bottom axis) and interfacial tunneling width (*w*, top axis) for (**e**) BDS, (**f**) NDS, and (**g**) TDS. The output current exhibits an apparent linear correlation with *w* (indicated by solid lines and R^2^ values over 0.96) and a potential exponential dependence on Φ_B-right_ (indicated by shaded trends).

To establish a definitive structure–property relationship bridging interfacial configuration and electron transport, we initially evaluated the predictive capability of intrinsic molecular descriptors across these datasets under fixed electrode conditions. Electronic structure analysis indicates the molecular LUMO plays a central role in governing interfacial energy alignment. As depicted in Figure 3c, correlating the junction current with the *E*_LUMO_ reveals an exponential increase across all metal electrodes for the oligoacene series. However, when extending this framework to heteroaromatic systems incorporating terminal selenophene rings—specifically benzo-diselenophene (BDS), naphtha-diselenophene (NDS), and tetra-diselenophene (TDS) (Figure 4a)—the junction current in strongly chemisorbed regimes deviates markedly from this exponential scaling. The molecular structures and corresponding energy-level alignments are shown in Figure S4. This breakdown occurs primarily because replacing terminal fused benzene rings with more reactive selenophene units induces intricate interfacial dipole interactions and pronounced orbital hybridization upon chemisorption, thereby disrupting the linear correspondence between the Schottky barrier height and the isolated molecular LUMO level.

In addition, we also investigated how varying the electrode material influences interfacial charge transport for a given molecular species within the framework of classical Schottky theory [20]. According to this model, the output current is expected to be exponentially suppressed by the barrier height: *I* ∝ exp(−Φ_B_). To understand how electrode characteristics dictate this barrier and the resulting transport, we first examined the direct effect of the pristine metal work function on the junction current. As illustrated in Figure 3d, the output current displays a steep jump upon transitioning from noble metals (physisorption) to reactive metals (chemisorption), with current levels in the physisorption regime being significantly lower than those in the chemisorption regime. By incorporating the molecular LUMO levels and interfacial dipole distributions, we further evaluated the dependence of the output current on the Schottky barrier height at the left contact (Φ_B-left_, Figure 3e). To examine the generality across diverse contact regimes and interfacial coupling strengths, we additionally investigated Pd(111), Ni(111), and Al(111) electrodes to evaluate the influence of varying electrode work functions and hybridization degrees on Φ_B-left_ and the resulting transport current. Intriguingly, for a given molecule, the junction current increases as the Schottky barrier rises, directly contradicting the conventional predictions of Schottky barrier theory.

To account for the dual-interface nature of the two-probe junction, we further evaluated the output current as a function of the right-contact Schottky barrier height (Φ_B-right_). As illustrated in Figure 3f, in sharp contrast to the trend observed for Φ_B-left_, the junction current exhibits an opposite dependence on Φ_B-right_—decreasing progressively as the barrier height increases. However, Φ_B-right_ alone still fails to establish a universal quantitative correlation with the output current across all systems. Furthermore, we also evaluated the absolute barrier asymmetry between the two contacts (|ΔΦ_B_| = |Φ_B-left_ − Φ_B-right_|), which is usually employed for the π-bridged molecule diodes [46]. The results reveal a distinct linear scaling with the output current across all molecular lengths (Figure 3g). However, the fitting coefficients exhibit moderate values ranging from R^2^ = 0.54 to 0.78, with smaller molecules such as benzene showing notably reduced linearity compared to extended conjugated frameworks.

From the aforementioned descriptor analysis, several critical insights emerge. Intrinsic molecular or metal properties in isolation are insufficient to predict interfacial charge transport across diverse contacts. Moreover, the conventional Schottky barrier height alone fails to adequately describe conduction across nanoscale metal/molecule/metal junctions. Critically, a thorough inspection of the physisorbed and chemisorbed transport configurations reveals that, alongside the energy barrier, the contact distance at the right interface plays a decisive role in governing transport variations. For instance, although chemisorbed systems exhibit larger substrate work functions and higher barrier heights than physisorbed ones, their substantially shorter interfacial separations enhance electronic tunneling, ultimately yielding significantly larger junction current.

Motivated by this dual dependence on spatial separation and barrier height, we performed a comprehensive evaluation grounded in Landauer transport theory that incorporates both interfacial quantum tunneling and thermal excitation effects [47]. Leveraging the Jeffreys–Wentzel–Kramers–Brillouin (JWKB) approximation, the electron tunneling probability across the interface *P*_TB_ can be estimated as follows [48]:

(2) $$P_{\mathrm{TB}}\;=\;\exp(-\frac{2w_{B}}{\hbar }\sqrt{2m\Phi_{B}})$$

where ℏ is the reduced Planck’s constant, *m* is the mass of the free electron, and *w* is the width of the square potential barrier. Following recent work [46], we adopt the comprehensive descriptor T = *w*^2^Φ_B_ to represent the interfacial tunneling probability. The larger T is, the smaller the output current becomes.

The decisive role of interfacial spatial separation is evidenced in Figure 3h, where the junction current displays a pronounced decaying dependence on the tunneling width *w* across all five acenes (R^2^ = 0.79–0.90). Particularly for smaller molecules, the relatively weak hybridization renders charge transport governed primarily by through-barrier tunneling, making *w* the dominant determining factor, as corroborated by the highest fitting coefficient reaching R^2^ = 0.90. Moreover, in physisorbed systems, although Cu(111) exhibits a higher Schottky barrier than Ag(111), its markedly shorter interface distance yields a lower overall tunneling descriptor T, leading to a higher transport current. Conversely, in chemisorbed systems, Pt(111) possesses the largest tunneling width despite having the lowest barrier, resulting in a larger T and reduced transmission.

As illustrated in Figure 3i, plotting ln *I* against the composite transmission parameter −T produces an exceptional linear scaling across all metal/molecule combinations, with correlation coefficients of R^2^ ≥ 0.90 (reaching up to 0.99 for Pc). Given that the PBE functional typically underestimates absolute LUMO levels due to self-interaction errors, we further benchmarked the frontier orbital energies using the hybrid HSE06 functional (Figure S5). We found that despite a systematic downward shift in absolute LUMO values, the relative energy scaling across the oligoacenes and the resulting linear correlations between ln*I* and −T (R^2^ > 0.90) are fully preserved. This confirms that the predictive capability and physical validity of our transmission parameter descriptor are robust against functional-dependent artifacts.

Furthermore, extending the molecular conjugated backbone systematically decreases the tunneling parameter T and boosts the output current. Crucially, when extending the π-conjugated series to larger hexacene (Hc) (the geometric structures are shown in Figure S6), the linear structure–property correlation is not only preserved (R^2^ = 0.94) but also exhibits a markedly steeper slope as the LUMO shifts downward, signifying an enhanced transport sensitivity to barrier variations in extended acenes. Moreover, we incorporated three additional electrode materials—Pd(111), Ni(111), and Al(111)—as an independent test set to validate the model’s generalizability. Geometric analyses (Figures S7–S9) confirm that the adsorption of oligoacenes on these metals is governed by strong chemisorption, characterized by pronounced molecular deformation. Remarkably, the descriptor predictions show outstanding quantitative agreement with first-principles transport calculations across all test substrates (R^2^ ≥ 0.90), successfully capturing the charge transport characteristics across both physisorbed and chemisorbed interfaces.

Based on the comprehensive calculations and analyses presented above, we have successfully constructed models for the adsorption structures of organic molecules of various sizes on metal surfaces and delved into the intrinsic relationship between these microscopic structures and interfacial electronic transport properties. Specifically, as shown in Figure S10, with the increase in the number of benzene rings and the elevation of the metal work function, we observed a notable enhancement trend in both the interfacial binding strength and electronic interaction. This change subsequently leads to a gradual reduction (enhancement) in the interfacial barriers (output current). Consequently, our work substantially enriches the interface database to enable rapid high-throughput screening of metal/molecule/metal junctions with targeted conductances for specific application scenarios.

### 2.5. Generality Validation of Our Descriptor

To verify the universality of descriptor T and the established transport mechanisms, we extended our investigation to heteroatom-containing systems (BDS, NDS, and TDS). Top views in Figure 4a reveal that the functionalized molecules uniformly prefer the bridge sites on these surfaces. Corresponding side views in Figure S11 further delineate two distinct interfacial interaction regimes. On coinage metals (Cu, Ag, Au), the molecules remain weakly physisorbed with large adsorption heights; despite the increasing molecular size, their conjugated backbones preserve flat, parallel configurations without noticeable distortion. Conversely, on transition metals (Pt, Rh, Ir), strong chemisorption and pronounced π–d hybridization dramatically shorten the adsorption distance, causing upward-tilted C–H bonds and a heavily corrugated molecular backbone that firmly anchors the molecule to the surface.

Figure 4b–d show the dependence of the junction current on the comprehensive transmission parameter −T across six transition metal electrodes (Cu, Ag, Au, Pt, Ir, and Rh) for BDS, NDS, and TDS, respectively. For all three diselenophene systems, the output current exhibits an evident exponential growth trend as −T increases, yielding high fitting goodness (R^2^ = 0.97 for BDS and NDS, and R^2^ = 0.91 for TDS). Across all cases, the noble metals (Cu, Ag, and Au) consistently populate the low-transmission regime with larger negative −T values, whereas the strongly hybridized reactive metals (Pt, Ir, and Rh) occupy the high-current regime, fully corroborating the predictive robustness of the combined transmission parameter T.

To further decouple the individual geometric and electronic contributions, we also individually analyzed the contributions of these two key factors (Table S2). As shown in Figure 4e–g, the output current is plotted against both the interfacial tunneling width (*w*, top axis) and the right-interface Schottky barrier height (Φ_B-right_, bottom axis). The junction current displays an apparent linear correlation with the interfacial tunneling width *w*, achieving exceptionally high linear fitting coefficients (R^2^ = 0.97 for BDS, R^2^ = 0.99 for NDS, and R^2^ = 0.96 for TDS). Concurrently, the junction current exhibits an apparent exponential-like decay with increasing Φ_B-right_ (indicated by the shaded regions). The data points deviating from this barrier-dependent trend are reconciled into the unified exponential scaling shown in Figure 4b–d through the cooperative contribution of the interfacial tunneling width. These results demonstrate that the dual governance of tunneling width and Schottky barrier height universally dictates the charge injection and electronic transport in metal/organic/metal Schottky junctions, even when heteroatoms are incorporated into the conjugated backbone.

## 3. Conclusions

In summary, we have systematically investigated the interfacial adsorption and quantum transport characteristics of prototypical acene molecules—ranging from Ben to Pc—on six transition metal surfaces (Ag, Cu, Au, Rh, Ir, and Pt) using first-principles calculations. The metal/organic interfaces are classified into distinct physisorbed and chemisorbed regimes according to their interaction nature. With increasing conjugated molecular size, the adsorption strength and electronic interactions intensify significantly. This evolution strengthens Fermi-level pinning, leading to a systematically decreasing slope in the linear scaling between the Schottky barrier height and the metal work function. Furthermore, by synergistically incorporating both the Schottky barrier height and the tunneling barrier width, we have established an interfacial descriptor T that correlates exponentially with transport current in practical circuit environments. Attaining a predictive accuracy above 90%, this descriptor markedly outperforms conventional Schottky theory and remains valid upon heteroatom substitution. Overall, our findings offer deep fundamental insights and establish a robust, predictive framework for designing and optimizing metal/organic/metal contacts in molecular and nanoscale electronic devices.

## 4. Methods

In this study, we utilized two distinct density functional theory (DFT) codes: the plane-wave-based Vienna Ab initio Simulation Package (VASP) [49] and the all-electron Fritz-Haber-Institute Ab initio Molecular Simulations (FHI-aims) [50]. Specifically, VASP was employed for structural optimization calculations, together with the Perdew-Burke-Ernzerhof (PBE) exchange-correlation functional [51]. To model the adsorption structures of Ben, Nc, Ac, Tc, and Pc molecules on the (111) surfaces of metals such as Ag, Cu, Au, Rh, Ir, and Pt, we constructed four-layer slabs with surface unit cells of varying sizes: (3 × 3), (4 × 3), (5 × 3), (6 × 3), and (7 × 3). The choice of a four-layer slab was benchmarked against thicker five- and six-layer models (Table S3), which demonstrated that adsorption energies vary by less than 0.04 eV (<5%) and work function shifts remain below 0.03 eV. Each slab was separated by a 30 Å vacuum region to ensure negligible interaction between the adsorbed molecule and its periodic images. To identify the most stable geometries of the organic molecules on metal surfaces, we considered four distinct sites: “top”, “bridge”, “fcc”, and “hcp”. In our calculations, the angles of the central C–C bonds relative to the close-packed metal orientations were set at 30°. During geometry optimizations, we constrained the bottommost metal layer while allowing the molecules and atoms in the three uppermost metal layers to relax. Structural optimizations were performed until the Hellmann–Feynman forces acting on all unconstrained atoms converged to less than −0.02 eV/Å in magnitude. The adequacy of this threshold was verified through benchmark calculations with criteria ranging from −0.01 to −0.04 eV/Å (Table S4), which yielded virtually identical total energies and adsorption configurations.

The stability of the adsorption systems is quantified by the adsorption energy, *E*_ads_, defined as *E*_ads_ = −(*E*_sys_ − *E*_sub_ − *E*_mole_). Here, *E*_sys_, *E*_sub_, and *E*_mole_ represent the total energies of the adsorption system, the clean metal substrate, and the organic molecule in the gas phase, respectively. To calculate the work functions, molecular dipole, and bond dipole potentials, a dipole correction is applied along the direction perpendicular to the metal surfaces. Using the optimized structures as a foundation, we calculated the electron potential energy of organic molecules adsorbed on metal surfaces with a 60 Å vacuum region included. For these calculations, a plane wave basis cutoff energy of 450 eV was employed. A Monkhorst-Pack grid [52] with a 3 × 5 × 1 *k*-point sampling was utilized for Brillouin zone integration. A convergence criterion of 1 meV/atom was imposed on the total energy to ensure the accuracy of our results. The adequacy of these settings was confirmed through systematic convergence tests (Table S5), which demonstrated that increasing the cutoff or *k*-point produces negligible energy fluctuations (<0.2 meV/atom and 0.7 meV/atom, respectively).

The FHI-aims code was utilized to compute the frontier orbital levels (HOMO and LUMO) of the isolated molecules, which were extracted from the calculated density of states (DOS) profiles and subsequently aligned to the absolute vacuum level via *E*_HOMO/LUMO_ = *ε*_HOMO/LUMO_ − *E*_vac_. *E*_vac_ represents the vacuum level (the individual DOS spectra and tabulated alignment data are detailed in Figure S12 and Table S6). The electronic density differences were obtained by subtracting the electron density of the isolated molecules and the clean metal surface from that of the adsorption systems: Δ*ρ*(r) = *ρ*_sys_(r) − *ρ*_sub_(r) − *ρ*_mole_(r). The planar-averaged charge density difference Δ*ρ*(z) was determined by integrating Δ*ρ*(r) over the in-plane x-y unit cell area: $\Delta \left(z\right)=\frac{1}{A}\iint \Delta \left(x,y,z\right)\mathrm{dxdy}$. To quantify interfacial charge rearrangement, the cumulative transferred charge was calculated as $Q\left(z\right)=\int_{-\infty }^{z}\Delta \left(z^{\prime }\right){\mathrm{dz}}^{\prime }$, which measures the net electron displacement across the plane at z and yields the effective interfacial charge transfer at its extremum. For these calculations, we employed the “tight” settings, which included the standard numerical atom-centered orbital basis sets. Specifically, we used the “tier2” basis sets for hydrogen (H) and carbon (C) and the “tier1” basis sets for sulfur (S), oxygen (O), selenium (Se), and copper (Cu). To ensure the accuracy of our results, we set the convergence thresholds as follows: 10^−2^ eV/Å for the final forces in structural relaxations, 10^−5^ electrons per unit volume for the charge density, and 10^−6^ eV for the total energy of the system.

The Atomistix Toolkit (ATK) R-2020.09-SP1 software, which leverages DFT combined with the Non-Equilibrium Green’s Function (NEGF) method, ref. [53] was utilized to calculate the electronic transport properties of metal–organic–metal systems. The computational models for these calculations comprised three distinct regions: the left electrode, the right electrode, and the central region, which incorporated the relaxed slab model. For the Schottky junction systems exhibiting physisorption, the distance between the right electrode and the metal substrate was fixed at twice the molecular average adsorption height observed in their respective systems. However, to facilitate comparisons among chemisorption systems, particularly focusing on the differences in transport properties caused by molecular deformation, we uniformly controlled the distance at twice the height of molecular chemisorption observed in the organic/Ir systems. Our calculations indicated it to be the maximum among all the chemisorbed systems. The I-V characteristics of both systems were computed using the Perdew-Burke-Ernzerhof (PBE) functional, with a double-ζ polarized Linear Combination of Atomic Orbitals (LCAO) basis set. For these calculations, we employed 261 *k*-points in the transport direction and set the real-space mesh cutoff to 80 Hartree (Ha).

## Data Availability

The original contributions presented in this study are included in the article and the Supplementary Information. Further inquiries can be directed to the corresponding authors.

## Supplementary Materials

The following supporting information can be downloaded at https://www.mdpi.com/article/10.3390/molecules31213848/s1. Figure S1. Schematic side views of adsorption configurations for benzene on Cu(111), Ag(111), Au(111), and Pt(111) surfaces based on the PBE+vdW^surf^−R method. White and orange spheres represent H and C atoms, respectively; dark green denotes Pt atoms, whereas gray collectively designates Cu, Ag, and Au atoms. The molecule adsorbs at the hcp hollow site on Cu, Ag, and Au(111) surfaces, whereas it prefers the bridge site on Pt(111) surface. Figure S2. Adsorption induced electron density redistribution integrated over the x-y plane (Δ*ρ*) for molecules on Ag(111) surface as a function of z-coordinate. Figure S3. The resulting total charge transfer (Q) for molecules on Ag(111) surface as a function of z-coordinate. Figure S4. Chemical structures of BDS, NDS, and TDS. The electronic energy levels of these molecules are shown in the right. Figure S5. (**a**) Gas-phase LUMO energy levels of the oligoacene series (Ben, Nc, Ac, Tc, and Pc) calculated using the HSE06 hybrid functional. (**b**) Linear correlations between ln*I* and −T obtained from the HSE06 calculations for the corresponding molecules. Solid lines represent linear fits, and R^2^ denotes the coefficient of determination. Figure S6. Side views of the optimized adsorption configurations of Hc on the four-layer (111) surface slabs of nine different metals (Ag, Au, Cu, Al, Ni, Pd, Rh, Ir, and Pt). For Ag and Au, the Hc molecule remains largely flat due to weak physisorption, whereas on Al, Ni, Pd, Rh, Ir, and Pt, the molecule exhibits noticeable bending and deformation, indicative of strong chemisorption or hybridization at the interface. Figure S7. Side views of the optimized adsorption configurations of Ben, Nc, Ac, Tc, and Pc on the four-layer Pd(111) surface slabs, modeled within (3 × 3), (4 × 3), (5 × 3), (6 × 3) and (7 × 3) surface supercells, respectively. Figure S8. Side views of the optimized adsorption configurations of Ben, Nc, Ac, Tc, and Pc on the four-layer Ni(111) surface slabs, modeled within (3 × 3), (4 × 3), (5 × 3), (6 × 3) and (7 × 3) surface supercells, respectively. Figure S9. Side views of the optimized adsorption configurations of Ben, Nc, Ac, Tc, and Pc on the four-layer Al(111) surface slabs, modeled within (3 × 3), (4 × 3), (5 × 3), (6 × 3) and (7 × 3) surface supercells, respectively. Figure S10. Schematic diagram illustrating the structure-activity relationship between the number of benzene rings, metal work function, and Schottky barrier as well as binding energy at the metal-organic interface. Figure S11. Side views of BDS, NDS, and TDS physisorption on the Ag/Cu/Au(111) surface and chemisorption on the Pt/Ir/Rh(111) surface. Figure S12. Calculated density of states (DOS) of the target molecular systems (Ben, Nc, Ac, Tc, Pc). The Fermi level is set to zero ($E_{F}=0\;\mathrm{eV}$). The numerical labels above the peaks indicate the respective positions (in eV) of the valence band maximum (VBM) and conduction band minimum (CBM), from which the vacuum-referenced frontier orbital energies are evaluated. Table S1. The calculated work functions (Φ_M_) of different metal surfaces, changes in work functions (ΔΦ_M_) induced by the adsorption of Ben, Nc, Ac, Tc, Pc molecules, molecular dipoles (*ϕ*^mole^), interfacial dipoles (*ϕ*^BD^) and Schottky barriers (Φ_B_). Table S2. The calculated work functions changes (ΔΦ_M_) induced by the adsorption of BDS, NDS and TDS molecules, molecular dipoles (*φ*^mole^), interfacial dipoles (*φ*^BD^), Schottky barriers (Φ_B_) and tunneling widths (*w*). Table S3. Calculated adsorption energies (*E*_ads_) and work function (Φ_M_) (in eV) for Ben adsorbed on various metal electrodes (Ag, Cu, Au, Rh, Ir, and Pt) with different slab thicknesses (4, 5, and 6 layers). Table S4. Convergence benchmark of the total energy per atom (*E*_total/atom_, in eV) for various transition metals (Ag, Cu, Au, Rh, Ir, and Pt) under different force convergence criteria. Table S5. Convergence test of the total energy per atom (*E*_total/atom_) with respect to the plane-wave cutoff energy and k-point sampling for various transition metals (Ag, Cu, Au, Rh, Ir, and Pt). Table S6. Calculated electronic properties, including ε_CBM_, ε_VBM_, vacuum level ($E_{\mathrm{vac}}$), and vacuum-aligned frontier orbital energy levels (*E*_LUMO_ and *E*_HOMO_), for the investigated molecules (all values in eV).
