# Supplementary material for: Insights into the Structural-Transport Property Relationship of the Size-Dependent Organic Molecules on Metal Surfaces
Source: Molecules. 2026 Oct 25;31(21):3848. doi: 10.3390/molecules31213848 (PMC13765177; doi:10.3390/molecules31213848)
Supplement: Supplementary file 1 [file molecules-31-03848-s001.zip › molecules-4600992-supplementary.pdf]

Supporting Information

Insights into the Structural-Transport Property Relationship of the Size-Dependent Organic Molecules on Metal Surfaces

Guirong Su[1,2,*], Yong Fan[3], Shuguang Zhang[1,2] and Yonghao Zhao[1,2,*]

[1] Jiangsu Provincial Engineering Research Center for Structure-Function Integrated Metallic Materials for Harsh Environments, School of Materials Science and Engineering, Hohai University, Changzhou, 213200, China

[2] Innovation Center for Critical Materials in Hydraulic Infrastructure Safety and Water Environment Restoration, School of Materials Science and Engineering, Hohai University, Changzhou, 213200, China

[3] School of Intelligent Agricultural Equipment Engineering, Shandong University of Technology, Zibo, 255000, China

Corresponding Author: grsu@hhu.edu.cn; yonghaozhao@hhu.edu.cn

Figure S1. Schematic side views of adsorption configurations for benzene on Cu(111), Ag(111), Au(111), and Pt(111) surfaces based on the PBE+vdW^{surf}–R method. White and orange spheres represent H and C atoms, respectively; dark green denotes Pt atoms, whereas gray collectively designates Cu, Ag, and Au atoms. The molecule adsorbs at the hcp hollow site on Cu, Ag, and Au(111) surfaces, whereas it prefers the bridge site on Pt(111) surface.

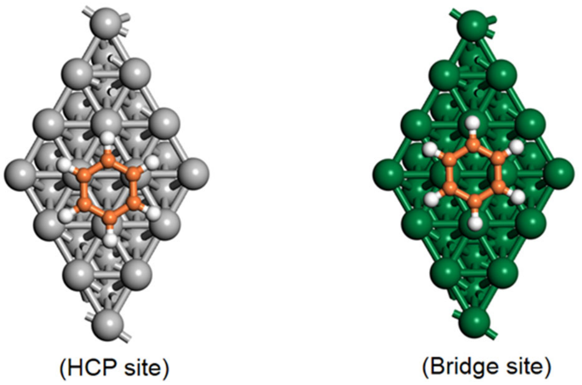

Figure S2. Adsorption induced electron density redistribution integrated over the *x*-*y* plane ($\Delta\rho$) for molecules on Ag(111) surface as a function of *z*-coordinate.

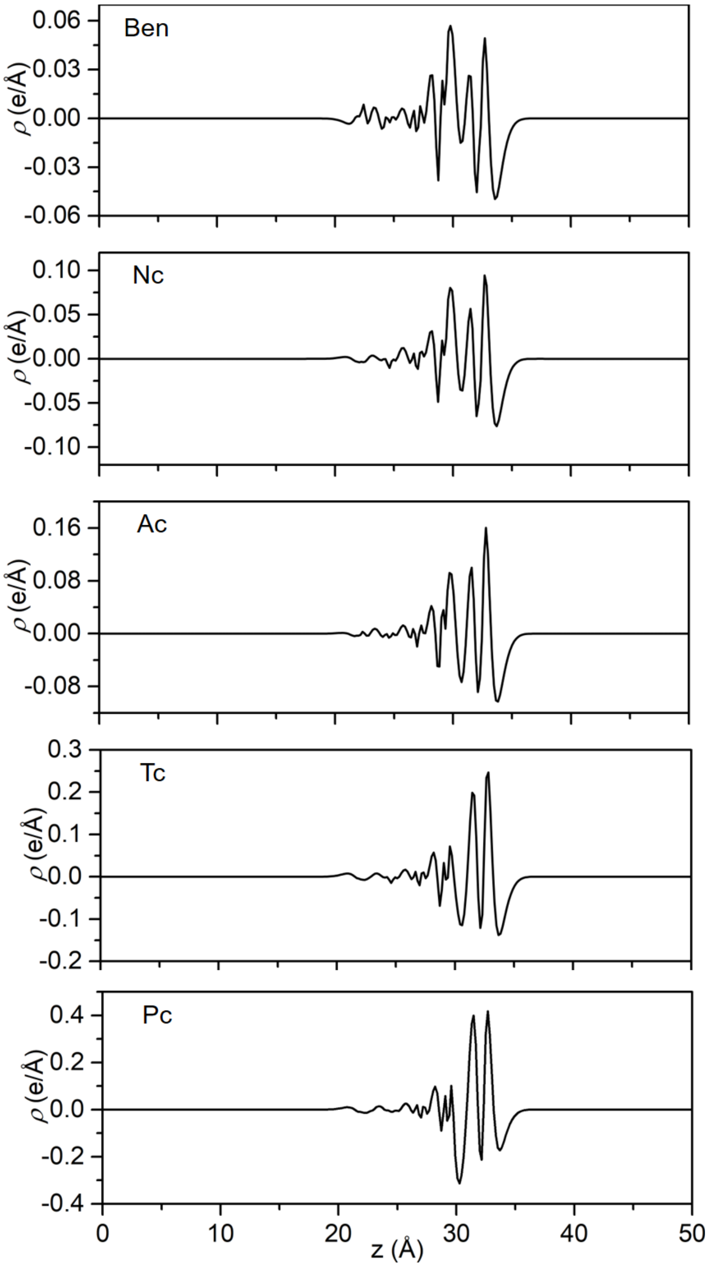

Figure S3. The resulting total charge transfer (Q) for molecules on Ag(111) surface as a function of z-coordinate.

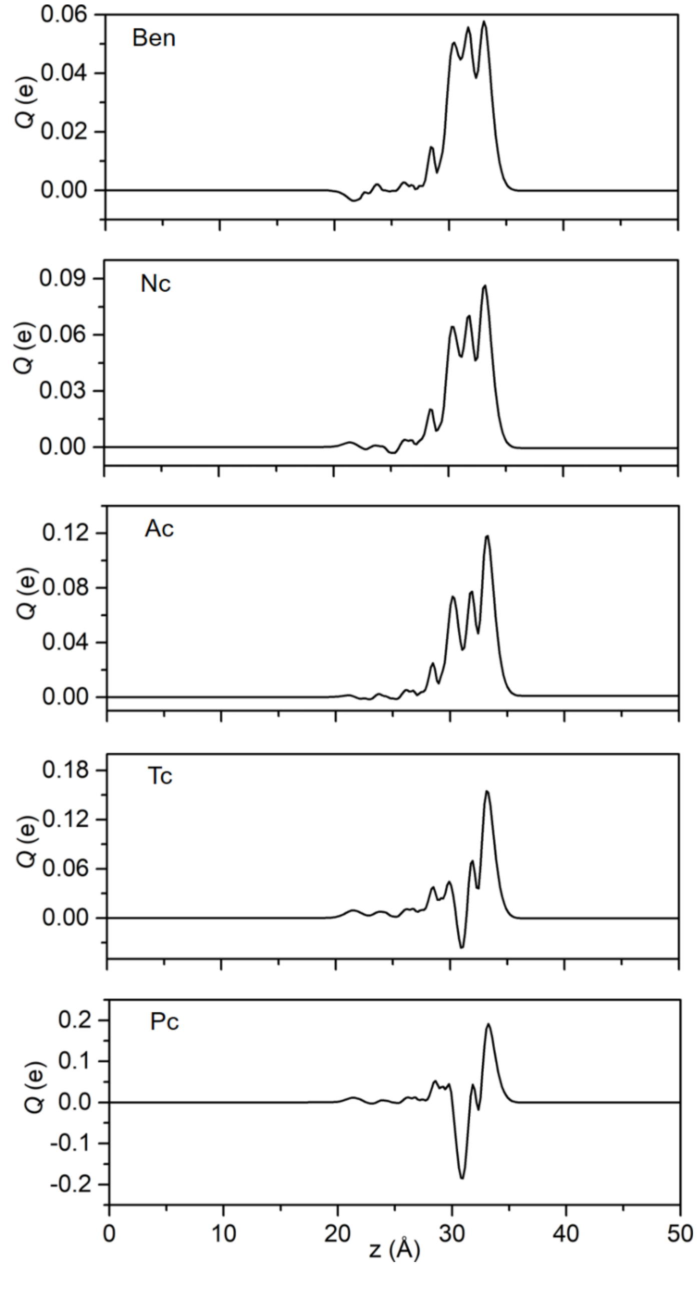

Figure S4. Chemical structures of BDS, NDS, and TDS. The electronic energy levels of these molecules are shown in the right.

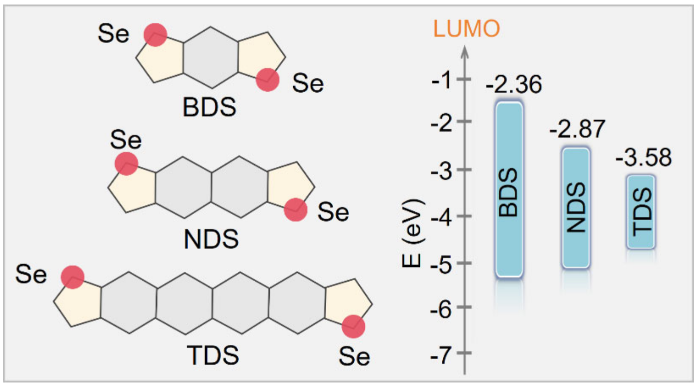

Figure S5. (a) Gas-phase LUMO energy levels of the oligoacene series (Ben, Nc, Ac, Tc, and Pc) calculated using the HSE06 hybrid functional. (b) Linear correlations between ln*I* and −T obtained from the HSE06 calculations for the corresponding molecules. Solid lines represent linear fits, and R^2 denotes the coefficient of determination.

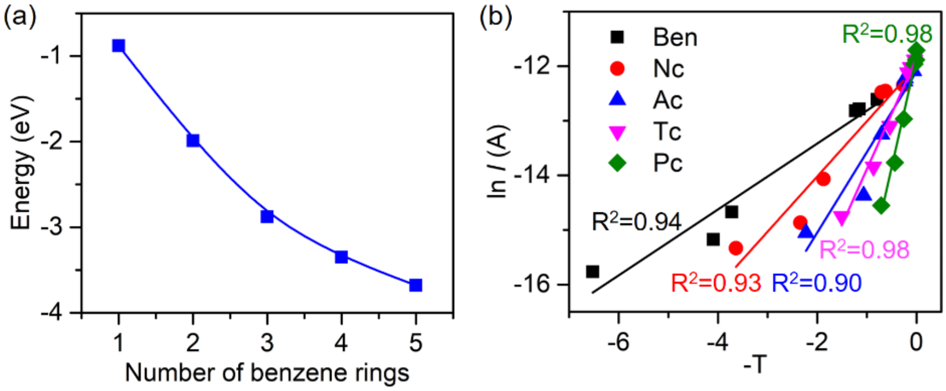

Figure S6. Side views of the optimized adsorption configurations of Hc on the four-layer (111) surface slabs of nine different metals (Ag, Au, Cu, Al, Ni, Pd, Rh, Ir, and Pt). For Ag and Au, the Hc molecule remains largely flat due to weak physisorption, whereas on Al, Ni, Pd, Rh, Ir, and Pt, the molecule exhibits noticeable bending and deformation, indicative of strong chemisorption or hybridization at the interface.

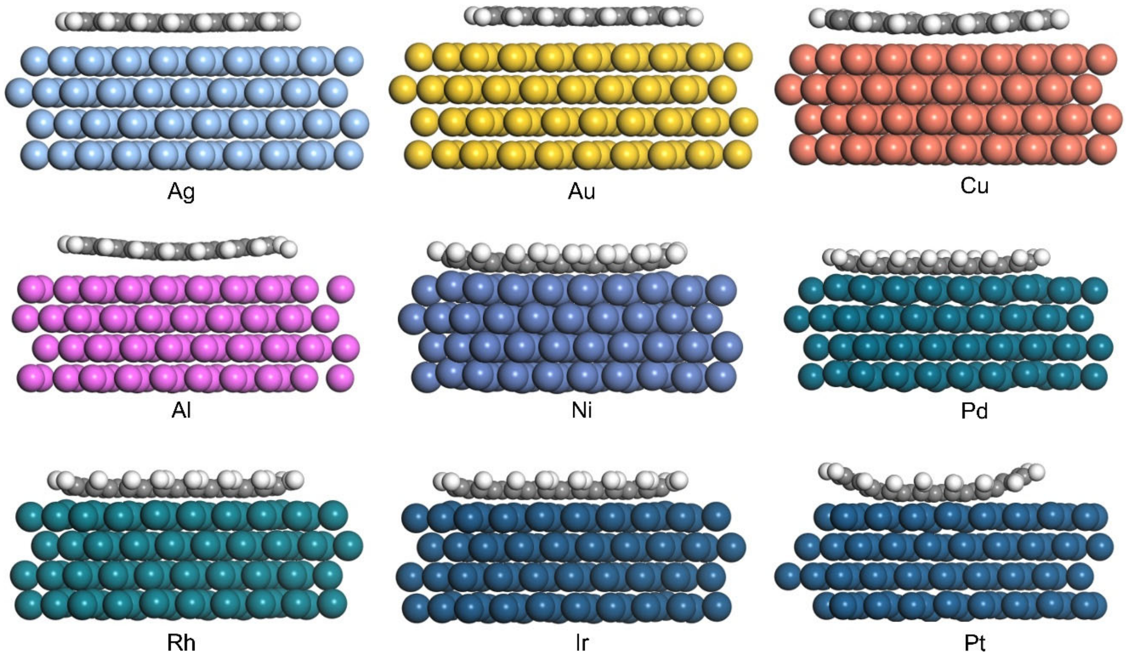

Figure S7. Side views of the optimized adsorption configurations of Ben, Nc, Ac, Tc, and Pc on the four-layer Pd(111) surface slabs, modeled within (3×3), (4×3), (5×3), (6×3) and (7×3) surface supercells, respectively.

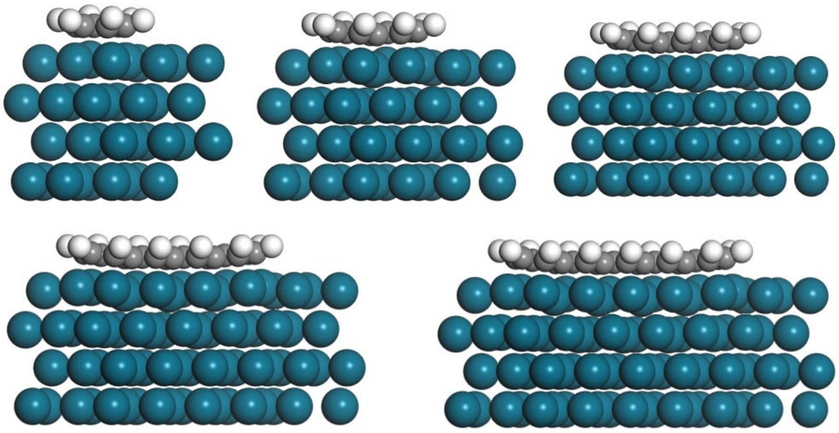

Figure S8. Side views of the optimized adsorption configurations of Ben, Nc, Ac, Tc, and Pc on the four-layer Ni(111) surface slabs, modeled within (3×3), (4×3), (5×3), (6×3) and (7×3) surface supercells, respectively.

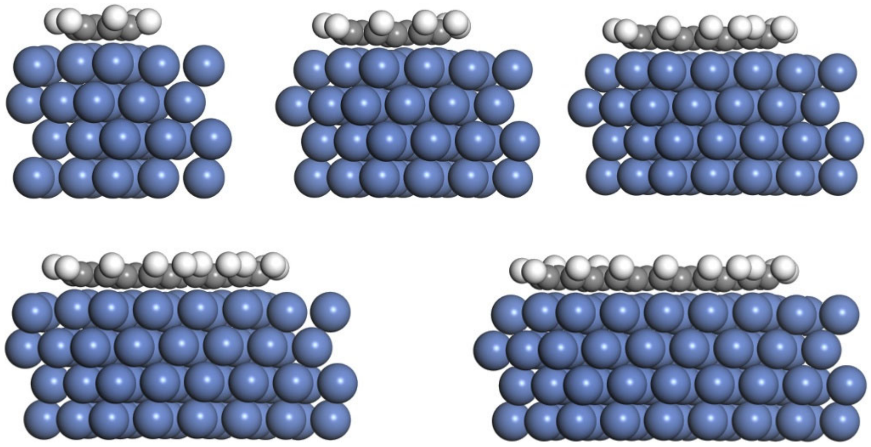

Figure S9. Side views of the optimized adsorption configurations of Ben, Nc, Ac, Tc, and Pc on the four-layer Al(111) surface slabs, modeled within (3×3), (4×3), (5×3), (6×3) and (7×3) surface supercells, respectively.

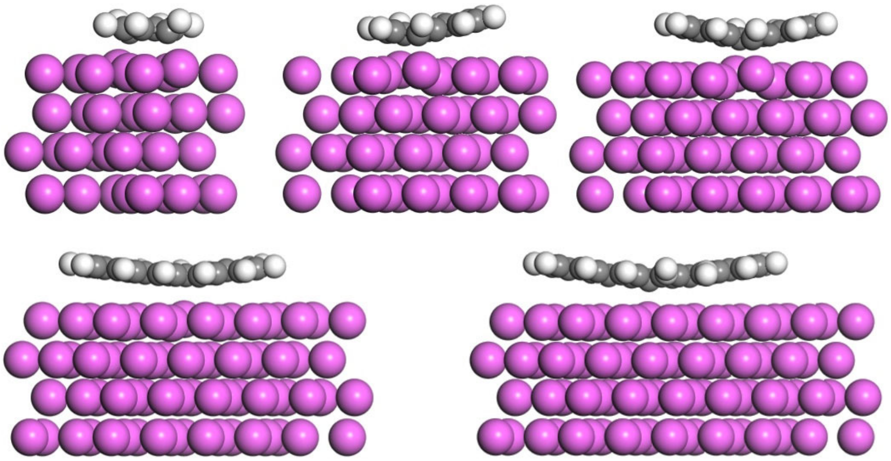

Figure S10. Schematic diagram illustrating the structure-activity relationship between the number of benzene rings, metal work function, and Schottky barrier as well as binding energy at the metal-organic interface.

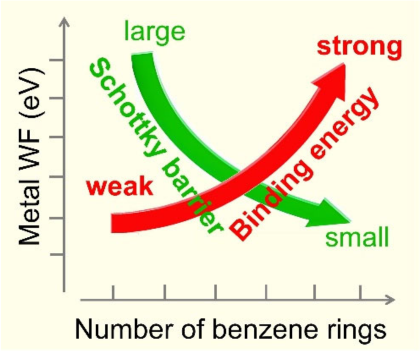

Figure S11. Side views of BDS, NDS, and TDS physisorption on the Ag/Cu/Au(111) surface and chemisorption on the Pt/Ir/Rh(111) surface.

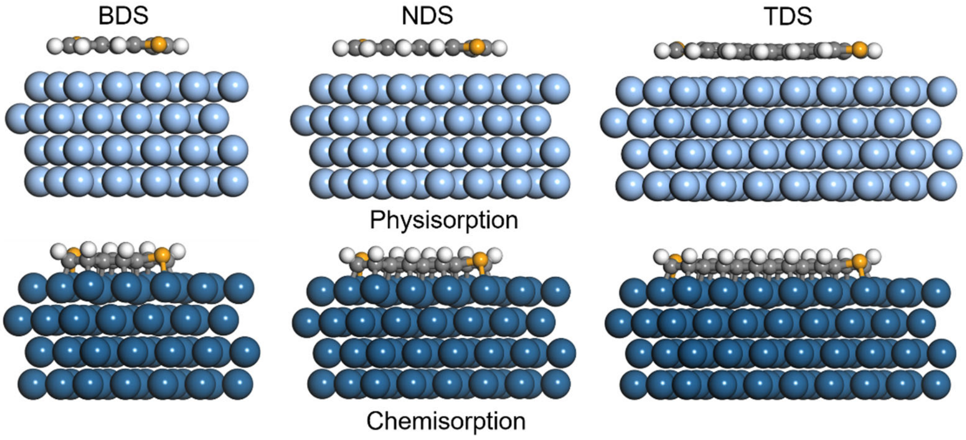

Figure S12. Calculated density of states (DOS) of the target molecular systems (Ben, Nc, Ac, Tc, Pc). The Fermi level is set to zero (E_F=0 eV). The numerical labels above the peaks indicate the respective positions (in eV) of the valence band maximum (VBM) and conduction band minimum (CBM), from which the vacuum-referenced frontier orbital energies are evaluated.

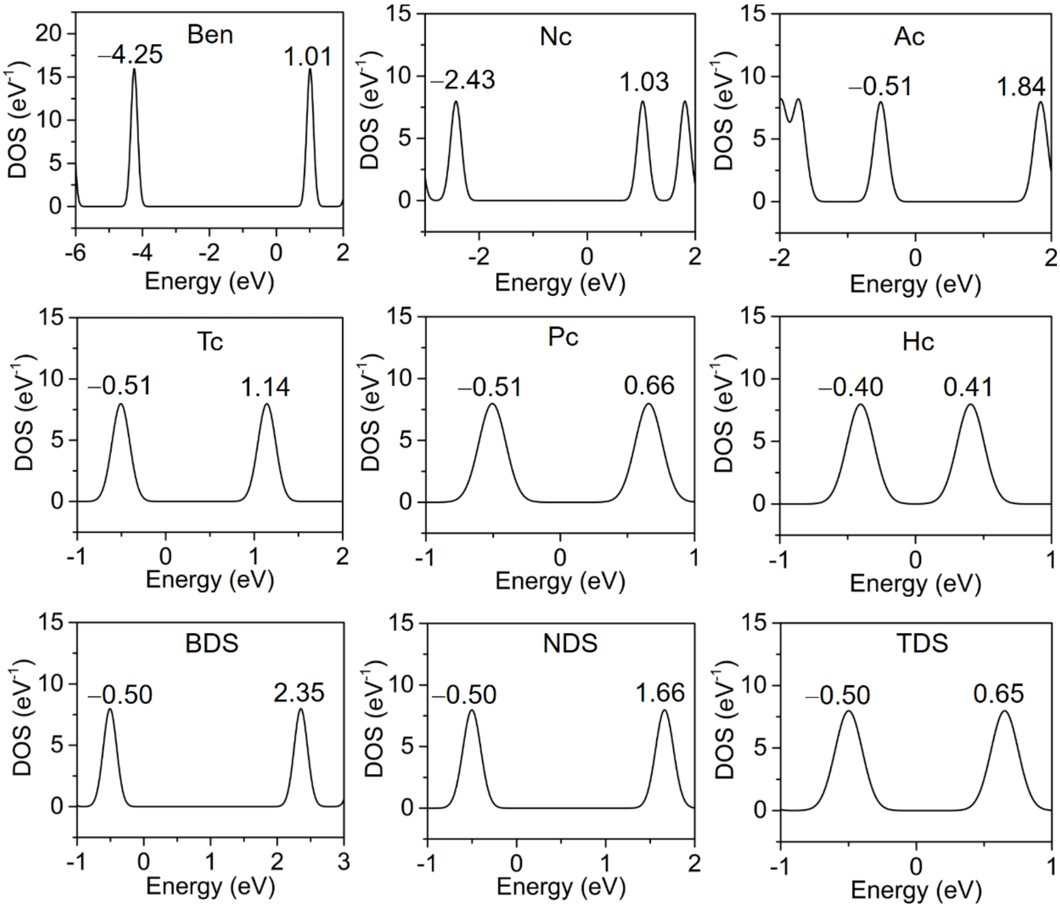

Table S1. The calculated work functions (Φ_M) of different metal surfaces, changes in work functions ($\Delta\Phi_M$) induced by the adsorption of Ben, Nc, Ac, Tc, Pc molecules, molecular dipoles (ϕ^{mole}), interfacial dipoles (ϕ^{BD}) and Schottky barriers (Φ_B).

Ben	**Ag**	**Cu**	**Au**	**Rh**	**Ir**	**Pt**
Φ_M	4.44	4.83	5.20	5.27	5.59	5.75
$\Delta\Phi_M$	−0.77	−0.91	−0.74	−1.48	−1.75	−1.87
ϕ^{mole}	0.02	0.02	0.02	−0.82	−0.92	−0.84
ϕ^{BD}	−0.79	−0.93	−0.76	−0.66	−0.83	−1.03
Φ_B	2.17	2.42	2.96	3.13	3.28	3.24
Nc	**Ag**	**Cu**	**Au**	**Rh**	**Ir**	**Pt**
$\Delta\Phi_M$	−0.80	−1.13	−0.76	−1.62	−1.95	−2.12
ϕ^{mole}	0.03	0.01	0.02	−0.90	−1.02	−0.97
ϕ^{BD}	−0.83	−1.14	−0.78	−0.72	−0.93	−1.15
Φ_B	1.13	1.21	1.94	2.07	2.18	2.12
Ac	**Ag**	**Cu**	**Au**	**Rh**	**Ir**	**Pt**
$\Delta\Phi_M$	−0.78	−1.11	−0.78	−1.54	−1.87	−2.17
ϕ^{mole}	0.02	0.17	0.03	−0.94	−1.07	−1.03
ϕ^{BD}	−0.8	−0.94	−0.81	−0.6	−0.8	−1.14
Φ_B	0.54	0.79	1.29	1.57	1.69	1.51
Tc	**Ag**	**Cu**	**Au**	**Rh**	**Ir**	**Pt**
$\Delta\Phi_M$	−0.68	−0.92	−0.77	−1.51	−1.87	−2.19
ϕ^{mole}	0.02	0.17	0.03	−0.94	−1.09	−1.07
ϕ^{BD}	−0.7	−0.75	−0.80	−0.57	−0.78	−1.12
Φ_B	0.23	0.57	0.89	1.19	1.3	1.12
Pc	**Ag**	**Cu**	**Au**	**Rh**	**Ir**	**Pt**
$\Delta\Phi_M$	−0.48	−1.01	−0.75	−1.46	−1.87	-2.23
ϕ^{mole}	−0.01	−0.16	0.04	−0.95	−1.12	−1.1
ϕ^{BD}	−0.47	−0.85	−0.79	−0.51	−0.75	−1.13
Φ_B	0.18	0.19	0.62	0.97	1.05	0.83

Table S2. The calculated work functions changes ($\Delta\Phi_M$) induced by the adsorption of BDS, NDS and TDS molecules, molecular dipoles (ϕ^{mole}), interfacial dipoles (ϕ^{BD}), Schottky barriers (Φ_B) and tunneling widths (w).

BDS	**Ag**	**Cu**	**Au**	**Rh**	**Ir**	**Pt**
$\Delta\Phi_M$	−0.9	−1.22	−1.12	−0.22	−0.43	−0.46
ϕ^{mole}	0.07	0.11	0.04	−0.97	−1.07	−1.02
ϕ^{BD}	−0.97	−1.33	−1.16	0.75	0.64	0.56
Φ_B	3.1	3.79	4	2.09	2.51	2.77
w	2.34	2.3	2.18	1.12	1.12	1.09
NDS	**Ag**	**Cu**	**Au**	**Rh**	**Ir**	**Pt**
$\Delta\Phi_M$	−0.79	−1.14	−0.84	−0.24	−0.46	−0.57
ϕ^{mole}	0.03	0.08	0.02	−0.98	−1.08	−1.04
ϕ^{BD}	−0.82	−1.22	−0.86	0.74	−0.62	−0.47
Φ_B	2.47	3.26	3.24	1.52	2.04	2.39
w	2.5	2.07	2.53	1.14	1.23	1.12
TDS	**Ag**	**Cu**	**Au**	**Rh**	**Ir**	**Pt**
$\Delta\Phi_M$	−0.7	−0.96	−0.76	−0.25	−0.53	−0.71
ϕ^{mole}	0.04	0.02	0.04	−0.54	−1.11	−1.08
ϕ^{BD}	−0.74	−0.98	−0.8	0.29	0.58	0.37
Φ_B	1.68	2.31	2.42	1.31	1.35	1.51
w	2.59	2.23	2.67	1.1	1.19	1.78

Table S3. Calculated adsorption energies (E_{ads}) and work function (Φ_M) (in eV) for Ben adsorbed on various metal electrodes (Ag, Cu, Au, Rh, Ir, and Pt) with different slab thicknesses (4, 5, and 6 layers).

E_{ads}	**Ag**	**Cu**	**Au**	**Rh**	**Ir**	**Pt**
4L	0.72	0.79	0.79	2.56	2.24	2.1
5L	0.74	0.82	0.80	2.53	2.27	2.13
6L	0.75	0.83	0.81	2.59	2.28	2.15
Φ_M	**Ag**	**Cu**	**Au**	**Rh**	**Ir**	**Pt**
4L	4.44	4.83	5.2	5.27	5.59	5.75
5L	4.46	4.84	5.22	5.28	5.60	5.77
6L	4.47	4.88	5.21	5.28	5.61	5.78

Table S4. Convergence benchmark of the total energy per atom ($E_{total/atom}$, in eV) for various transition metals (Ag, Cu, Au, Rh, Ir, and Pt) under different force convergence criteria.

EDIFFG	**Ag**	**Cu**	**Au**	**Rh**	**Ir**	**Pt**
−0.01	−4.1148	−4.7356	−4.2494	−7.2758	−8.3175	−6.3615
−0.02	−4.1148	−4.7356	−4.2494	−7.2758	−8.3175	−6.3615
−0.03	−4.1148	−4.7356	−4.2494	−7.2758	−8.3175	−6.3615
−0.04	−4.1148	−4.7356	−4.2494	−7.2758	−8.3175	−6.3615

Table S5. Convergence test of the total energy per atom ($E_{total/atom}$) with respect to the plane-wave cutoff energy and k-point sampling for various transition metals (Ag, Cu, Au, Rh, Ir, and Pt).

Encut (eV)	**Ag**	**Cu**	**Au**	**Rh**	**Ir**	**Pt**
370	–4.1154	–4.7400	–4.2546	–7.2756	–8.3179	–6.3615
390	–4.1152	–4.7381	–4.2543	–7.2758	–8.3179	–6.3615
410	–4.1152	–4.7369	–4.2544	–7.2760	–8.3179	–6.3617
430	–4.1150	–4.7356	–4.2542	–7.2758	–8.3175	–6.3615
450	–4.1148	–4.7356	–4.2542	–7.2758	–8.3175	–6.3615
470	–4.1148	–4.7358	–4.2540	–7.2756	–8.3175	–6.3615
490	–4.1148	–4.7358	–4.2540	–7.2756	–8.3175	–6.3613
k-points	**Ag**	**Cu**	**Au**	**Rh**	**Ir**	**Pt**
3×1×1	–4.1625	–4.5773	–4.0979	–7.1227	–8.1581	–6.2265
3×2×1	–4.1077	–4.7242	–4.2417	–7.2663	–8.3075	–6.3740
3×3×1	–4.1238	–4.7421	–4.2542	–7.2750	–8.3163	–6.3663
3×4×1	–4.1158	–4.7352	–4.2506	–7.2756	–8.3175	–6.3627
3×5×1	–4.1148	–4.7356	–4.2494	–7.2758	–8.3175	–6.3615
3×6×1	–4.1146	–4.7360	–4.2496	–7.2756	–8.3175	–6.3608
3×7×1	–4.1144	–4.7363	–4.2500	–7.2756	–8.3173	–6.3606

Table S6. Calculated electronic properties, including ε_{CBM}, ε_{VBM}, vacuum level (E_{vac}), and vacuum-aligned frontier orbital energy levels (E_{LUMO} and E_{HOMO}), for the investigated molecules (all values in eV).

	**Ben**	**Nc**	**Ac**	**Tc**	**Pc**	**Hc**	**BDS**	**NDS**	**TDS**
ε_{CBM}	1.01	1.03	1.84	1.14	0.66	0.41	2.35	1.66	0.65
ε_{VBM}	-4.25	-2.43	-0.51	-0.51	-0.51	-0.40	-0.50	-0.50	-0.50
E_{vac}	2.49	3.50	5.12	4.82	4.62	4.90	4.72	4.54	4.24
E_{LUMO}	-1.48	-2.47	-3.28	-3.68	-3.96	-4.49	-2.37	-2.88	-3.59
E_{HOMO}	-6.74	-5.93	-5.63	-5.33	-5.13	-5.30	-5.22	-5.04	-4.74
